# Control of Disturbing Loads in Residential and Commercial Buildings via Geometric Algebra

**DOI:** 10.1155/2013/463983

**Published:** 2013-10-23

**Authors:** Manuel-V. Castilla

**Affiliations:** Higher Polytechnic School, University of Seville, 41011 Seville, Spain

## Abstract

Many definitions have been formulated to represent nonactive power for distorted voltages and currents in electronic and electrical systems. Unfortunately, no single universally suitable representation has been accepted as a prototype for this power component. This paper defines a nonactive power multivector from the most advanced multivectorial power theory based on the geometric algebra (GA). The new concept can have more importance on harmonic loads compensation, identification, and metering, between other applications. Likewise, this paper is concerned with a pioneering method for the compensation of disturbing loads. In this way, we propose a multivectorial *relative quality index* $\tilde{\delta }$ associated with the power multivector. It can be assumed as a new index for power quality evaluation, harmonic sources detection, and power factor improvement in residential and commercial buildings. The proposed method consists of a single-point strategy based of a comparison among different relative quality index multivectors, which may be measured at the different loads on the same metering point. The comparison can give pieces of information with magnitude, direction, and sense on the presence of disturbing loads. A numerical example is used to illustrate the clear capabilities of the suggested approach.

## 1. Introduction

In recent years, there has been a growing concern for power system distortion. This is due in part to a proliferation of nonlinear loads such as air conditioning equipment, computers, fax and copying equipment, and printers, extensively used in commercial buildings, that are using the same power network as fluorescent lighting and various electronic communications equipment. The result of using such highly nonlinear load is that the current waveform is distorted, causing excessive harmonic voltages to be generated. The close proximity of many of these commercial buildings (hotels, offices, departmental stores, shopping centres, and hospitals) will definitely contribute to the distortion of the electric power quality of the feeder supplying these buildings.

With such high cost of poor power quality, researchers have developed so many technical solutions to eliminate or at least to reduce the impacts of poor power quality on modern buildings. Such solutions consist of the design of passive and active filters as well as designing switching regulators for computer power supplies. However, to install such power quality correction devices, people working in the building industry must first be aware of the problem and appreciate the cost of the problem as well as knowing the cost of the solutions. They should also be aware of the power quality components and the control for each of these components. The main aim of this research work is to define those aspects that directly influence the power quality of modern commercial buildings so that building designers can be aware of the challenges required in such buildings. Once they are aware of the problem, the decision to install or not install correction devices could be clearly made. The harmful and costly effects of harmonics have been discussed extensively [1, 2] and spurred stringent requirements by international institutions regarding the allowed levels of harmonics at the point of connection to the power supply [3, 4].

The increase of distortion levels caused by faults and switching events in the residential and commercial loads affects significantly the power transfer quality of a supply. As is well known, harmonic presence can have adverse effects on the linear/nonlinear operation of power systems. Existing indexes [5] reflect the degree of power disturbance but fail to assess the precise form of all the above mentioned phenomena together. In this paper, a relative quality index multivector $\left(\tilde{\delta }\right)$ is suggested to evaluate the non-active power in *n-*sinusoidal systems in stationary conditions. This has the advantage of evaluating the power quality at any selected single-point strategy in a distribution network by means of this multivectorial indicator. The conventional quantities based on the concepts of active, reactive, and apparent powers cannot provide any useful information with regard to the harmonic power flow. 

From a theoretical point of view, in a circuit of any topology, with sinusoidal or *n*-sinusoidal supply voltage, the *apparent power *(*S*) may be analytically decomposed into two components [6]. These components are the *active power *(*P*) and the *non-active power *(*Q*_*F*_), which sum to the apparent power by the relationship *S* ^2^ = *P* ^2^ + *Q*_*F*_^2^. In sinusoidal conditions, *non-active power* has the only value *Q*_*F*_ = *Q*_1_ = *U*_1_ *I*_1_sin⁡⁡ *φ*_1_, and complete compensation is possible so as to render the overall power factor to be the unity (ideal condition), and in this case, *S* = *P*⇒*Q*_1_ = 0. On the contrary, many definitions have been formulated to represent non-active power for *n*-sinusoidal situations in linear and nonlinear power systems. In fact, the physical reality is that *P* ≤ *S*, but there, as sinusoidal case, is not physical justification, in general, for separating the difference *Q*_*F*_^2^ = *S* ^2^ − *P* ^2^ or non-active power into analytical quantities.

Reference [7] argues that “*any such decomposition of S is entirely mathematical and subject to the whims and interpretations of individuals.”* In this sense, in *n*-sinusoidal operation, the presence of harmonic distortion on voltage and currents generates a number of power terms that involve the non-active power. It is our conviction that apparent power concept should be represented by a set of adaptable orthogonal terms to any association criteria. Starting with the works of Budeanu and Fryze, numerous valuable works have appeared [8–11] aimed to characterize the power equation into different components, among them reactive and distortion powers, with diverse names and meanings without a powerful reason for it. But none of these have succeeded in defining a concept that not only accounts for the total non-active power but also satisfies a multivectorial representation. The discussion on this matter is still open, and there is not yet a generalized power theory that can be assumed as a common base for power quality evaluation and harmonic source detection. 

It is well known that the distorted current causes disturbances on the supply side, because of the nonzero impedance of the source. The load side of the power system also is affected by the distorted currents. Therefore, for a generic metering point, harmonic sources can be located on the utility side and/or costume side, so that both supply and/or load may be responsible for the distortion origin. In the literature, several different theories for harmonic sources detection have been suggested, but the most common method is the “power direction method” [12] that checks the direction of harmonic power flow. However, in some applications this approach is not appropriate to provide correct information about the source producing harmonic distortion. Furthermore, some approaches have been proposed for evaluation and detection harmonic sources. They can be mainly classified in two groups, *single-point* and *multipoint* measurement methods [13]. Both techniques have their advantages and disadvantages.

Unfortunately, in some practical applications, these approaches cannot provide correct information about the customer and the supply side harmonic contributions. Nevertheless, we must recognize that *single-point method* presents many advantages as simple instrumentation, low cost, and easy implementation. For all this, we believe that a good solution to the problem can be the measure of a multivectorial index with its three attributes, magnitude, direction, and sense, which carries all necessary information. This index is based on the power multivector concept [14].

The purpose of this paper is to suggest a relative quality index, with the inherent ability to simultaneously register two types of information in residential and commercial loads:

- detection and control of the dominant harmonic source;
- optimal compromise between power factor and power quality.

The key ideas of the approach are the use of the Clifford algebras (GA) to obtain the power multivector. From this, it is possible to define the $\tilde{\delta }$ quantity.

## 2. Mathematical Foundations

Geometric algebra is a mathematical structure, developed over the last 40 years, based on Clifford algebras. A geometric algebra can be defined simply by specifying appropriate rules for multiplying vectors. Thus, let *𝒱* ^*n*^ be an *n*-dimensional linear space over the real numbers. The geometric product of vectors *a* ⊗ *b*, *a*, *b* ∈ *𝒱* ^*n*^, can be decomposed into a symmetric inner product

(1) $$\begin{array}{c} a\cdot b=\frac{1}{2}\left(a⊗b+b⊗a\right) \end{array}$$

and an antisymmetric outer product

(2) $$\begin{array}{c} a\wedge b=\frac{1}{2}\left(a⊗b-b⊗a\right). \end{array}$$

Therefore, *ab* has the canonical decomposition:

(3) $$\begin{array}{c} a⊗b=a\cdot b+a\wedge b. \end{array}$$

**Figure 1:**
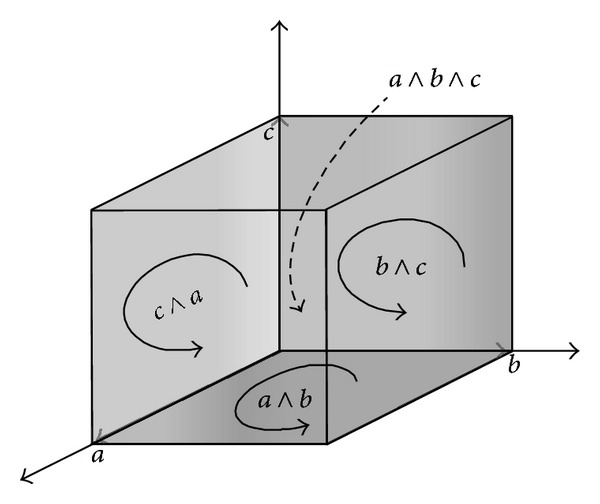
Representation of geometric objects: vector, bivector, and trivector.

The inner product *a* · *b* is a scalar, and the outer product *a* ∧*b* is called a bivector (or 2-vector). Geometrically, it represents a directed plane, in much the same way as a vector represents a directed line segment (Figure 1). We can regard *a*∧*b* as a directed area with a norm ||*a*∧*b*|| equal to the usual scalar area of each parallelogram in Figure 1, with the direction of the plane in which the parallelogram lies, whose sense can be assigned to the parallelogram in the plane. Therefore, just as a vector *a*∧*b* represents a directed plane segment, a trivector (3-vector) *a*∧*b*∧*c* represents a directed space segment (the parallelepiped with edges *a*, *b*, and *c*).

However, the electrical quantities, voltage and current, have no easy interpretation in classic Clifford algebra. For a clear representation of these waveforms, a new geometric algebra has been constructed—a generalization of the classic Clifford algebra—which we have termed “*Generalized Complex Geometric Algebra”* denoted by {*𝒞𝒢*_*n*_, ⊙} [15]. In this framework, *𝒞* is the complex vector space, *𝒢*_*n*_ is the Clifford algebra or geometric algebra associated with the *n*-dimensional real space *𝒱* ^*n*^, and ⊙ : (*ℜ*∘⊗) is the new *generalized geometric product*. A detailed description of this new structure, of the geometric product “⊙”, and their properties is given as $\hat{g}$ in [15].

## 3. Power Multivector: Control Strategy

### 3.1. Power Multivector

In classic circuit theory, apparent power volt-amperes  *S* is defined as the product of rms voltage and rms current at the circuit terminals. In order to represent the concept of the power multivector in *𝒞𝒢*_*n*_, we introduce a new rule for multiplying geometric phasors (complex vectors). This rule is the new geometric product ⊙ : (*ℜ*∘⊗), where *ℜ* is an application over complex planes and “⊗” is the classic geometric product. 

**Figure 2:**
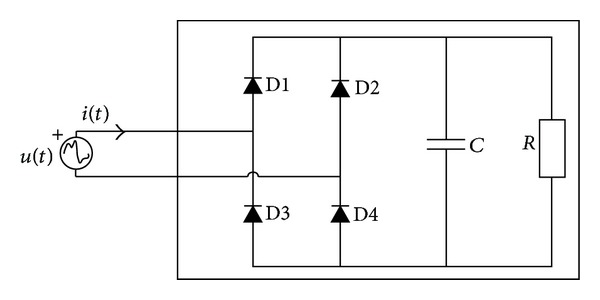
Nonlinear circuit with *n*-sinusoidal waveforms.

In this context, consider an arbitrary non-linear one-port circuit, Figure 2, supplied by the voltage:

(4) $$\begin{array}{c} u\left(t\right)=\sqrt{2}\sum_{p\in L\cup N}U_{p}\;\sin\left(p\omega t+\alpha_{p}\right). \end{array}$$

The resulting current has an instantaneous value given by

(5) $$\begin{array}{c} i\left(t\right)=\sqrt{2}\sum_{q\in N\cup M}I_{q}\;\sin\left(q\omega t+\beta_{q}\right), \end{array}$$

where *q* is the harmonic order of *i*(*t*). Figure 2 shows the circuit of a diode rectifier with a capacitive output filter which is an example of a nonlinear load with harmonic voltage source behaviour. This kind of circuit is present in almost all residential and commercial loads, such as computers, video monitors, TV sets, and electronic lamp ballasts [16]. 

In the {*𝒞𝒢*_*n*_, ⊙} structure spanned by an orthonormal basis {*σ*_1_, *σ*_2_, *σ*_3_,…, *σ*_*n*_}, the associated *p*th harmonic voltage and *q*th harmonic current can be represented by the geometric-phasors:

(6) $$\begin{array}{c} {\tilde{U}}_{p}=U_{p}e^{j\;\alpha_{p}}\sigma_{p},\;\;{\tilde{I}}_{q}=I_{p}e^{j\beta_{q}}\sigma_{q}, \end{array}$$

where $U_{p}=||{\tilde{U}}_{p}||$ , $I_{q}=||{\tilde{I}}_{q}||$ .

Apparent power can thus be expressed as a multivector $\tilde{S}$ in the geometric algebra (*n*-sinusoidal case) in a parallel way to the apparent power $\overset{-}{S}$ in the complex algebra (sinusoidal case). In this sense, the generalised geometric product of the voltage and conjugate current geometric-phasors is given by

(7) $$\begin{array}{c} \tilde{S}=\tilde{U}⊙{\tilde{I}}^{*}=\sum_{\begin{array}{c} p\in N\cup L\\ q\in N\cup M \end{array}}^{}{\tilde{U}}_{p}⊙{\tilde{I}}_{q}^{*}=\left\{\underset{{\tilde{\Omega }}^{•}}{\underset{︸}{\tilde{U}\cdot {\tilde{I}}^{*}}}+\underset{{\tilde{\Omega }}^{\wedge }}{\underset{︸}{\tilde{U}\wedge {\tilde{I}}^{*}}}\right\}, \end{array}$$

which obeys the usual conservation law [13] assuming that a group of voltage harmonics *N* exist that have corresponding current harmonics of the same frequencies, that components *L* of the supply voltage exist without corresponding currents and that components *M* of current exist without corresponding voltages. From (7), one can easily define a complex-scalar part $\left({\tilde{\Omega }}^{•}\right)$ and a complex-bivector part $\left({\tilde{\Omega }}^{\wedge }\right)$ where, for clarity of presentation and without loss of generality, *α*_*p*_ = *α*_*q*_ = 0. In this particular case, *ℜ* is the identity, and then, the scalar $\left({\tilde{\Omega }}^{•}\right)$ and the bivector $\left({\tilde{\Omega }}^{\wedge }\right)$ are now given by

(8) $$\begin{array}{c} {\tilde{\Omega }}^{•}=\sum_{p\in N}U_{p}I_{p}e^{j\varphi_{p}}\sigma_{0}, \end{array}$$

(9) Ω~∧=∑p<qp,q∈N(UpIqejφq−UqIpejφp)σpq+∑p∈L∪N, q∈Mp∈L, q∈NUpIqe−jβqσpq.

It follows from (8) that

(10) Re{Ω~•}=∑p∈NUpIpcos⁡⁡φpσ0,

(11) Im⁡{Ω~•}=∑p∈NUpIpsin⁡φpσ0,

where ||Re{Ω~•}|| is the *active power P* and ||Im⁡{Ω~•}|| is associated with Budeanu and Slonim's *reactive power Q*.

The imaginary part of the complex scalar Im⁡{Ω~•} and the complex bivector ${\tilde{\Omega }}^{\wedge }$ have a non-independent physical nature and constitute the nonactive power multivector $\tilde{\Sigma }$ , which is defined as

(12) Σ~=jQσ0+∑p<qp,q∈N(UpIqejφq−UqIpejφp)σpq+∑p∈L∪N, q∈Mp∈L, q∈NUpIqe−jβqσpq.

The squared value ${||\tilde{S}||}^{2}$ in (7) may be represented as

(13) ||S~||2=||U~  ⊙  I~∗||2=||U~||2||I~||2=S2,

(14) ||S~||2=||P||2+||Σ~||2=S2.

Equations (13) and (14) are identical to the classic squared value of the apparent power and are only a secondary result of this work. In particular, $S^{2}={||\tilde{S}||}^{2}$ is the sum of the squared magnitudes of the individual components of $\tilde{S}$ . In this way, in numerous situations, direction and sense are not required; the magnitude $||\tilde{S}||=S$ was then established to manage a specific problem. However, there are situations where direction and sense are necessary, as those that depend on the origin and nature of distortion. In these cases, the power multivector $\tilde{S}$ is an appropriate quantity to solve the problem since it incorporates all the required information within one single succinct expression.

The convenience of using one or another quantity is dictated by the necessities of the situation. Particularly, in sinusoidal case, (6) can be expressed by

(15) $$\begin{array}{c} \tilde{U}=\left|{\tilde{U}}_{1}\right|e^{j\alpha_{1}}\sigma_{1}={\overset{-}{U}}_{1}\sigma_{1},\;\;\tilde{I}=\left|{\tilde{I}}_{1}\right|e^{j\beta_{1}}\sigma_{1}={\overset{-}{I}}_{1}\sigma_{1}, \end{array}$$

where ${\overset{-}{U}}_{1}$ and ${\overset{-}{I}}_{1}$ are now the Steinmetz *classic phasors*. Using (7), the complex power is defined by

(16) $$\begin{array}{c} \tilde{S}=\tilde{U}⊙{\tilde{I}}^{*}=\left(P+jQ\right)\sigma_{0}, \end{array}$$

where *P* = *U*_1_ *I*_1_cos⁡ *φ*_1_ and *Q* = *U*_1_ *I*_1_sin⁡*φ*_1_  are the *active* and *reactive* powers, respectively. In addition, the power multivector representation under *n*-sinusoidal operation can be perceived as a generalization of the complex power representation valid only in 1-sinusoidal operation.  Table 1 shows the correspondence between both approaches.

### 3.2. Control Strategy: Relative Quality Index

From the viewpoint of the power factor improvement residential and commercial loads, the suggested representation (7) can be particularly useful. Thus, a multivectorial relative quality index $\tilde{\delta }$ is defined by

(17) δ~=S~P~=1+jQ~BP~+Ω~∧P~⇒||δ~||2=1+||∑p=qUpIpsin⁡φp||2||P~||2+||∑p≠qΩ~pq∧||2||P~||2,

and the *power factor *(PF) can be written as

(18) $$\begin{array}{c} \text{PF}=\frac{1}{||\tilde{\delta }||}, \end{array}$$

where

(19) $$\begin{array}{c} ||\tilde{\delta }||=\sqrt{1+\frac{{||\tilde{Q}||}^{2}}{{||\tilde{P}||}^{2}}+\frac{{||{\tilde{\Omega }}^{\wedge }||}^{2}}{{||\tilde{P}||}^{2}}}. \end{array}$$

From the viewpoint of the disturbing loads control, (17) shows that on $\tilde{\delta }$ all their terms, with direction and sense, are accessible to any compensation strategy. This property is important in order to achieve an appropriated compromise between any quality index and the power factor. First, $\tilde{S}=\tilde{P}\Rightarrow ||\tilde{\delta }||=1$ can be considered as an ideal condition. Thus, the *relative quality index *unity is a minimum reference value, since it corresponds to a case on absence of non-active power. 

On the other hand, ${||\tilde{S}||}^{2}-{||\tilde{P}||}^{2}={||{\tilde{Q}}_{F}||}^{2}$ is ( $||{\tilde{Q}}_{F}||$ Fryze's reactive power) is the maximum value for the non-active power. It is obvious that $||{\tilde{Q}}_{F}||$ depends on both common and uncommon harmonics of the voltage and current. Then, the different $\tilde{\delta }$ multivectors and their magnitudes depend on the supply voltage and load conditions. Each situation is strictly related to the distortion state of power system and, therefore, to the harmonic presence and power quality. Thus, a higher amount of non-active power is associated with a higher contribution of the harmonics to the ${\tilde{Q}}_{F}$ power terms. In this case, two pieces of information exists.

- From $\tilde{\delta }$ , it is possible to obtain a very good power factor through an adequate compensation strategy. Evidently, $\tilde{\delta }$ can register the bidirectional active power harmonic components and can efficiently handle situations in which a bidirectional active power flow occurs.
- If $||\tilde{\delta }||$ is more significant, a higher contribution of the non-active power harmonics occurs. On the contrary, if $||\tilde{\delta }||$ is small, the influence of the harmonic contribution is reduced.

From these considerations, an evaluation of both $\tilde{\delta }$ and $||\tilde{\delta }||$ , calculated in the same point metering and in the same working conditions, could give two pieces of information on the concrete problems of the power quality and control of disturbing loads.

## 4. Numerical Example

Let us consider that a building is supplied from a source of periodical *n*-sinusoidal voltage with a geometric phasor given by

(20) $$\begin{array}{c} \tilde{U}=200e^{j0}\sigma_{1}+200e^{-j30}\sigma_{3}+100e^{j30}\sigma_{5}\left(\text{V}\right). \end{array}$$

The load impedance tensor building is given by

(21) 𝒵Load=(41.57+j30.9300059.64+j107.23000157.74+j238.23),

and the resulting building current geometric phasor is given by

(22) I~L=3.86e−j36.65°σ1+1.63e−j90.92°σ3 +0.35e−j26.49°σ5(A)⇒||I~L||=4.21(A).

### 4.1. Without Compensation

According to (7), the following power multivector is obtained:

(23) S~L=(797.14+j774.91)σ0+(−311.21−j382.82)σ13   +(−320.75−j13.48)σ15+(−93.11−j6.9)σ35(VA),

where

(24) Q~L=j774.91σ0(VA),Ω~13L∧=(−311.21−j382.82)σ13(VA),Ω~15L∧=(−320.75−j13.48)σ15(VA),  Ω~35L∧=(−93.11−j6.9)σ35(VA).

It follows that $||{\tilde{S}}_{L}||=1261.4\;\left(\text{VA}\right),\;P_{L}=\;797.14\;\left(\text{W}\right)$ , $||{\;\tilde{\Omega }}_{L}^{\wedge }||=595.97\;\left(\text{VA}\right)$ , $||{\tilde{\delta }}_{L}||=1.58$ , and PF_*L*_ = 0.63.

Figure 4(a) shows the scalar and bivector parts of the multivector ${\tilde{S}}_{L}$ .

### 4.2. Passive Partial Compensation

**Figure 3:**
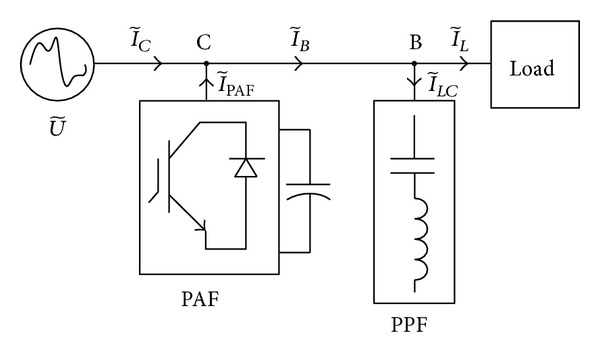
Architecture of the compensator.

With regard to the power factor improvement, consider first that an *LC*-branches shunt (parallel passive filter, PPF), Figure 3, is connected to the load terminals to fully compensate the load harmonic susceptances according to [17]. The selected poles are

(25) $$\begin{array}{c} p1=1.2\omega_{0},\;\;p2=4\omega_{0},\\ p3=6\omega_{0}\left(\omega_{0}=100\;\pi \left(\text{rad}/\text{s}\right)\right). \end{array}$$

Simulation results for parameters to the PPF are

(26) L1=733.96 mH,  C1=9.59 μF,L2=227.19 mH,  C2=2.79 μF,L3=124.58 mH,  C3=2.26 μF.

In this way, we obtain the current geometric phasor and load power multivector in point *B* given by

(27) I~B=3.1ej(0°)σ1+0.79ej(−30°)σ3 +0.19ej(30°)σ5(A)⇒||I~B||=3.21(A),S~B=(797+j0)σ0+(−400.1−j231)σ13 +(−235.56+j136)σ15+(−20.5+j35.5)σ35(VA).

It follows that

(28) ||S~B||=961.41(VA),  PB=797(W),      QB=0(VA),  ||Ω~13B∧||=462(VA),||Ω~13B∧||=272(VA),  ||δ~B||=1.20,  PFB=0.83.

**Figure 4:**
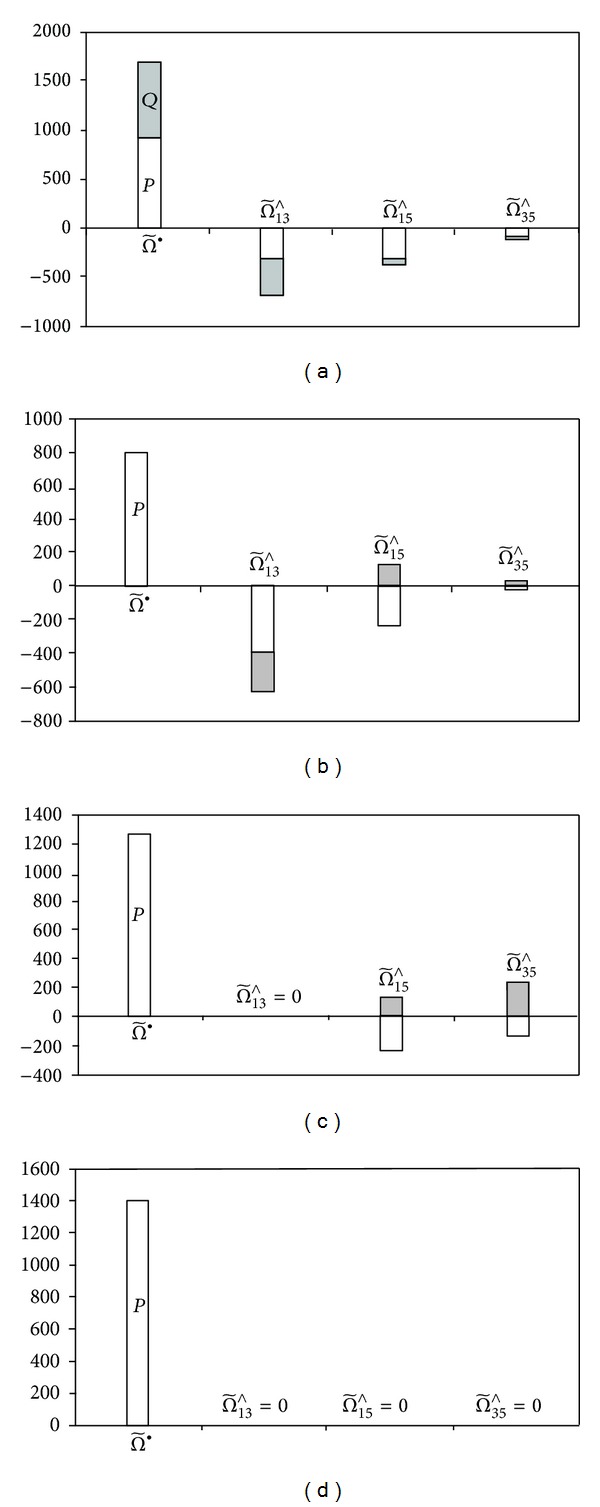
Apparent power components (□ real part, ■ imaginary part): (a) without compensation, (b) PPF compensation, (c) selective PAF-PPF compensation, and (d) total PAF-PPF compensation.

Figure 4(b) illustrates the scalar and bivector parts of the multivector ${\tilde{S}}_{B}$ .

### 4.3. Selective Hybrid Compensation

In a second step, a selective compensation to the distortion power bivector is possible in order to obtain an optimal compromise between the relative quality index (19) and power factor; that is, $||{\tilde{\Omega }}_{13_{B}}^{\wedge }||=0\;\left(\text{VA}\right)$ . Without going into detail, the compensator would consist of a parallel active filter (PAF) producing a sinusoidal current. The PAF is controlled to modify the 3rd order harmonic of the ${\tilde{I}}_{B}$ current according to condition *U*_*p*_ *I*_*q*_ − *U*_*q*_ *I*_*p*_ = 0. The generated geometric phasor harmonic reference current and the corresponding power bivector part are given by ${\tilde{I}}_{3_{\text{PAF}}}=2.303e^{j(150^{{}^{\circ}})}\sigma_{3}\;\left(\text{A}\right)$ and ${\tilde{\Omega }}_{13_{\text{PAF}}}^{\wedge }=400.1+j231\;\left(\text{VA}\right)$ , respectively. After this selective compensation, the current geometric phasor and power multivector at point *C* are given by

(29) I~C=3.1ej(0°)σ1+3.1ej(−30°)σ3+0.19ej(30°)σ5(A),S~C=(1259+j0)σ0+(0+j0)σ13 +(−235.55+j136)σ15 +(−136+j235.55)σ35(VA),

where $||{\tilde{S}}_{C}||=1316.45\;\left(\text{VA}\right),\;P_{C}=1259\;\left(\text{W}\right)$ , *Q*_*C*_ = 0 (VA), $||{\tilde{\Omega }}_{13_{C}}^{\wedge }||=0\;\left(\text{VA}\right)$ , $||{\tilde{\Omega }}_{15_{C}}^{\wedge }||=272\;\left(\text{VA}\right)$ , and $||{\tilde{\Omega }}_{35_{C}}^{\wedge }||=272\;\left(\text{VA}\right)$ . Moreover, $||{\tilde{\delta }}_{C}||$ and PF_*C*_ are very close to unity; that is, $||{\tilde{\delta }}_{C}||=1.04$ and PF_*C*_ = 0.96. Scalar and bivector parts of the multivector ${\tilde{S}}_{C}$ are shown in Figure 4(c).

### 4.4. Total Hybrid Compensation

With regard to the total power factor improvement, that is, $||{\tilde{\Omega }}_{L}^{\wedge }||=0\;\left(\text{VA}\right)\Rightarrow \;\;\left(||{\tilde{\Omega }}_{13_{C}}^{\wedge }||=0,\;\;||{\tilde{\Omega }}_{15_{C}}^{\wedge }||=0,\;\;||{\tilde{\Omega }}_{35_{C}}^{\wedge }||=0\right)$ , *Q*_*L*_ = 0 (VA), and PF = 1, the same procedure can be used as for the previous case. In this way, the PAF is controlled to modify the 3rd and 5th harmonic of the ${\tilde{I}}_{B}$ current. The generated reference current geometric phasors and power bivector parts ${\tilde{\Omega }}_{13_{\text{PAF}}}^{\wedge }$ , ${\tilde{\Omega }}_{15_{\text{PAF}}}^{\wedge }$ , and ${\tilde{\Omega }}_{35_{\text{PAF}}}^{\wedge }$ are given by

(30) $$\begin{array}{c} {\tilde{I}}_{3_{\text{PAF}}}=2.303e^{j(150^{{}^{\circ}})}\sigma_{3}\left(\text{A}\right),\;\;{\tilde{I}}_{5_{\text{PAF}}}=1.36e^{j({-150}^{{}^{\circ}})}\sigma_{5}\left(\text{A}\right),\\ {\tilde{\Omega }}_{13_{\text{PAF}}}^{\wedge }=400.1+j231\left(\text{VA}\right), \end{array}$$

(31) $$\begin{array}{c} {\tilde{\Omega }}_{15_{\text{PAF}}}^{\wedge }=235.55-j136\left(\text{VA}\right),\\ {\tilde{\Omega }}_{35_{\text{PAF}}}^{\wedge }=136-j235.55\left(\text{VA}\right), \end{array}$$

respectively.

The current and power multivectors at point *C* are given by

(32) I~C=3.1ej(0°)σ1+3.1ej(−30°)σ3 +1.55ej(30°)σ5(A)⇒||I~C||=4.65(A),S~C=1395  σ0(VA),  ||δ~C||=PFC=1.

The effect of this total compensation is illustrated in Figure 4(d).

In cases *c* and *d*, selective and total compensation, power bivectors to the load and compensator are of the same nature despite their opposite signs in real and imaginary parts. It is necessary to note the importance of the direction and sense in the power multivector. 

This compensation strategy increases the active power in the same way as in [17] but has two advantages: the ability to adjust the reference current into PAF and no resonance problems. 

It is remarkable that the power factor is not the exclusive index for power quality and different aspects (economics, compensator architecture, power factor, power quality, etc.) may be relevant for the partial cancellation of the non-active powers. In this sense, as can be seen through the proposed example, an optimal compromise between power factor and power quality (case *c*) could require suitable power decompositions. 

Finally, through this numerical example, a formal procedure for power factor correction has been realized within the mathematical framework {*𝒞𝒢*_*n*_} using information exclusively contained in the power multivector concept. The suggested apparent power multivector, (7), can readily handle these cases, since it carries all information required and its superiority compared to any power equation is clearly demonstrated in the proposed example.

## 5. Conclusions 

This paper presents a new power multivector, which is universally applicable to systems with any kind of voltage and current waveforms as the residential and commercial loads. The basis of the theory is the use of the Clifford spaces, (GA), to define the power multivector as a direct sum of complex scalar and complex bivector components. Our multivector plays a similar role to that of the Steinmetz phasor model in the sinusoidal case. Our approach obeys the usual conservation laws [14] and is internally consistent with existing power equations. The application of these new concepts to power system analysis should make significant improvements possible in control devices, new optimization algorithms, and effective power quality indexes in residential and commercial loads.

## Figures and Tables

**Table 1:** 

*1*-sinusoidal case: complex power	*n*-sinusoidal case: power multivector
$\overset{-}{S}=\overset{-}{U}\cdot {\overset{-}{I}}^{*}$	$\tilde{S}=\tilde{U}⊙{\tilde{I}}^{*}=\tilde{U}\cdot {\tilde{I}}^{*}⊕\tilde{U}\wedge {\tilde{I}}^{*}={\tilde{\Omega }}^{•}⊕{\tilde{\Omega }}^{\wedge }$
$\overset{-}{S}=\overset{-}{U}\cdot {\overset{-}{I}}^{*}=P+jQ$	$\tilde{S}=\tilde{U}⊙{\tilde{I}}^{*}=(P+jQ)\sigma_{0}⊕{\tilde{\Omega }}^{\wedge }$

## References

[1] Wagner VE, Balda JC, Griffith DC (1993). Effects of harmonics on equipment. *IEEE Transactions on Power Delivery*.

[2] Henderson RD, Rose PJ (1994). Harmonics: the effects on power quality and transformers. *IEEE Transactions on Industry Applications*.

[3] Recommended Practices and Requirements for Harmonic Control in Power Systems.

[4] Limits for Harmonic Current Emissions.

[5] Sharon D, Montaño JC, Borras D, Castilla M, López A, Gutieerez J (2008). Power quality factor for networks supplying unbalanaced nonlinear loads. *IEEE Transactions on Instrumentation and Measurement*.

[6] Fryze S (1932). Wik-, Blind, un Scheinleitung in Elektrischen Stromkreisen mit nichtsinusoidalem Verlauf von Strom und Spanung. *Elektrotechnische Zeitschrift*.

[7] Shepherd W, Zakikhani P (1972). Suggested definition of reactive power for nonsinusoidal systems. *Proceedings of the Institution of Electrical Engineers*.

[8] Czarnecki LS (1997). Budeanu and Fryze: two frameworks for interpreting power properties of circuits with nonsinusoidal voltages and currents. *Electrical Engineering*.

[9] Sharon D (1973). Reactive power definitions and power factor improvement in nonlinear systems. *Proceedings of the Institution of Electrical Engineers*.

[10] Slonim MA, Van Wyk JD (1988). Powers components in a system with sinusoidal and nonsinusoidal voltages and/or currents. *IEE Proceedings B*.

[11] Emanuel AE (1990). Powers in nonsinusoidal situations a review of definitions and physical meaning. *IEEE Transactions on Power Delivery*.

[12] Xu W, Liu Y (2000). A method for determining customer and utility harmonic contributions at the point of common coupling. *IEEE Transactions on Power Delivery*.

[13] Muscas C (1996). Assessment of electric power quality and related problems. *ETEP*.

[14] Castilla M, Bravo JC, Ordóñez M (2008). Geometric algebra: a multivectorial proof of Tellegen’s theorem in multiterminal networks. *IET Circuits, Devices and Systems*.

[15] Castilla M, Bravo JC, Ordóñez M, Montano JC (2008). Clifford theory: a geometrical interpretation of multivectorial apparent power. *IEEE Transactions on Circuits and Systems I*.

[16] Ghorbani MJ, Rad MS, Mokhtari H, Honarmand ME, Youhannaie M Residential loads modeling by norton equivalent model of household loads.

[17] Czarnecki LS (1991). Scattered and reactive current, voltage, and power in circuits with nonsinusoidal waveforms and their compensation. *IEEE Transactions on Instrumentation and Measurement*.

